# Municipal investment in off-road trails and changes in bicycle commuting in Minneapolis, Minnesota over 10 years: a longitudinal repeated cross-sectional study

**DOI:** 10.1186/s12966-017-0475-1

**Published:** 2017-02-13

**Authors:** Jana A. Hirsch, Katie A. Meyer, Marc Peterson, Le Zhang, Daniel A. Rodriguez, Penny Gordon-Larsen

**Affiliations:** 1 Department of Epidemiology and Biostatistics, Arnold School of Public Health, University of South Carolina, 915 Greene Street, Columbia, SC 29208 USA; 2 Department of Nutrition, Gillings School of Global Public Health, University of North Carolina at Chapel Hill, 123 West Franklin Street, Chapel Hill, NC 27516 USA; 3 Carolina Population Center, University of North Carolina at Chapel Hill, Chapel Hill, NC USA; 4 Department of City & Regional Planning, University of North Carolina at Chapel Hill, Chapel Hill, NC USA; 5 New York City Department of City Planning, New York, NY USA; 6 Department of City & Regional Planning, University of California Berkeley, Berkeley, CA USA

**Keywords:** Bicycle commuting, Greenway, Built Environment, Physical Activity, Urban Planning, Urban Population

## Abstract

**Background:**

We studied the effect of key development and expansion of an off-road multipurpose trail system in Minneapolis, Minnesota between 2000 and 2007 to understand whether infrastructure investments are associated with increases in commuting by bicycle.

**Methods:**

We used repeated measures regression on tract-level (*N* = 116 tracts) data to examine changes in bicycle commuting between 2000 and 2008–2012. We investigated: 1) trail proximity measured as distance from the trail system and 2) trail potential use measured as the proportion of commuting trips to destinations that might traverse the trail system. All analyses (performed 2015–2016) adjusted for tract-level sociodemographic covariates and contemporaneous cycling infrastructure changes (e.g., bicycle lanes).

**Results:**

Tracts that were both closer to the new trail system and had a higher proportion of trips to destinations across the trail system experienced greater 10-year increases in commuting by bicycle.

**Conclusions:**

Proximity to off-road infrastructure and travel patterns are relevant to increased bicycle commuting, an important contributor to overall physical activity. Municipal investment in bicycle facilities, especially off-road trails that connect a city’s population and its employment centers, is likely to lead to increases in commuting by bicycle.

**Electronic supplementary material:**

The online version of this article (doi:10.1186/s12966-017-0475-1) contains supplementary material, which is available to authorized users.

## Background

Despite widespread knowledge of the multitude of benefits, a majority of Americans still fail to reach recommended levels of physical activity [1, 2]. Active commuting-walking or biking to work-provides physical activity [3] with known health benefits [4], as well as decreased motorized vehicle traffic and emissions [5]. Building urban environments to facilitate active commuting behaviors, and thus physical activity, into daily routines has become a local and national priority. As of January 2016, over 800 municipalities passed Complete Streets policies to enable safe street access for all users, “including pedestrians, bicyclists, motorists and transit riders of all ages and abilities.” [6] In September 2015, the U.S. Surgeon General’s Call to Action, “Step It Up!,” promoted “walkable communities” as benefitting “people of all abilities, including those who run, bike, skate, or use wheelchairs” [7].

Over the past decade, proportion of workers who commute to work by active modes has surged. Nationwide, the percentage of commuters who walk increased 12% between 2007 and 2016 and the percentage of commuters who bicycle increased 50% [8]. Concurrently, the 50 most populous U.S. cities experienced more pronounced increases in active commuting, with 14% increase in pedestrian commuters and 71% increase in bicycling commuters [8]. However, overall rates remain low, with only 2.8 and 0.6% of U.S. commuters walking and bicycling, respectively, and 5.0 and 1.2% of the 50 most populous cities’ commuters walking and bicycling, respectively [8]. Given the low prevalence of bicycling, further efforts are crucial to understand factors shaping bicycle commuting.

Development of municipal cycling infrastructure has coincided with increased commuting rates. According to the Alliance for Bicycling & Walking 2016 Benchmarking Report, 36 states published goals to increase bicycling, up from 22 states in 2010, and 15 states established annual spending targets for bicycling and walking initiatives, up from 6 states in 2010 [8]. Similarly, 47 of the 50 most populous cities (up from 32 in 2010) have goals to increase bicycling activity [8]. These efforts seem to be working, as average miles of bicycle facilities per square mile in the 50 most populous cities doubled between 2007 and 2016 [8]. Identification of infrastructure investments that encouraged commuters to bicycle would better guide municipalities in their allocation of resources. While evidence exists for infrastructure effects nationally, many current studies are cross-sectional [9, 10]. Scarce longitudinal research has been published on this topic and has produced mixed results, potentially due to a limited time frame or differences in type of bicycle infrastructure investigated [11–17]. Furthermore, understanding infrastructure’s role requires consideration of not only proximity to new infrastructure but also potential commute patterns and accessibility to employment centers. This can be accomplished by investigating the proportion of work-related trips that might traverse the new infrastructure for some portion of the commuting route. Ultimately, off-road paved trails may be particularly conducive for encouraging bicycle commuting if they link residential areas with commercial and employment centers. In addition, off-road paved trails may encourage non-utility cycling by providing pleasant and safe cycling environments valued by leisure-cyclists [18].

We used data from Minneapolis, Minnesota to test whether increases in bicycle commuting between 2000 and 2010 are associated with implementation of an off-road trail system specifically designed to promote bicycle commuting. We hypothesized that increases in commuting by bicycle, measured at the census tract between 2000 and 2010, would be higher in tracts closer to the new off-road trails and in tracts with a higher proportion of work-related commuting trips that might benefit from the expanded trail networks.

## Methods

### Setting and study sample

This longitudinal repeated cross-sectional study used data from 116 tracts (based on 2010 Census boundary delineations) for Minneapolis. Among U.S. cities, Minneapolis represents an ideal city to understand the influence of bicycling infrastructure on commuting by bicycle. Of the 50 most populous U.S. cities, in the 2016 benchmarking report, Minneapolis had 3.9% of commuters who bicycled to work [8]- the third highest after Portland, Oregon and Washington, District of Columbia. This represents a 1.1 percentage point increase since 2007-the sixth largest increase across the most populous cities [8]. These increases correspond with investments and expansion in bicycle-oriented infrastructure since 2000. Between 2000 and 2010, Minneapolis, increased cycling infrastructure, resulting in 2.8 miles of cycling infrastructure per square mile in 2010 [19], and reaching 5.8 miles per square mile by 2016 [8]. The city’s cycling infrastructure included development of a 179.3 mile [8] off-road paved trail system designed to link the city’s population and its employment centers. Despite high investment in infrastructure in Minneapolis, to date no work has leveraged construction of new trails to examine longitudinal changes in bicycle commuting.

### Data sources

#### Bicycle commuting

Tract-level bicycle commuting was assessed with the Census 2000 and American Community Survey (ACS) 5-year average (2008–2012, representing 2010) question: “How did this person usually get to work last week?” If more than one method of transportation was used, respondents checked the mode used for most of the distance. We also included data on commuting by bicycle from the 1990 census to account for historical trends in cycling in our statistical analysis. We harmonized commute data to 2010 census boundaries using Brown University’s Longitudinal Tract Data Base (LTDB) methodology that uses areal interpolation, based on area- and population-weighting, to account for boundary changes (e.g., splits and consolidation) in tracts over time [20].

#### Built environment infrastructure

**Fig. 1:**
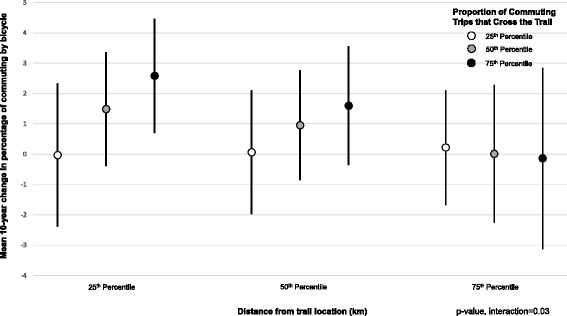
Multivariable-adjusted regression estimates^a^ for the difference^b^ in the percentage of workers commuting by bicycle in 2000 and 2010 according to joint levels^c^ of the distance (km) between the tract and the trail system^c^ and proportion of commuting trips that cross the trail system^d^. ^a^Regression models included: time-varying and tract-level variables for distance to trail system, proportion to work-related trips that cross the trail system, total work-related trips, intersection density, population density, median household income, professional workforce, workforce aged 13–34 years, total length of bicycle lanes, maximum reach of bicycle lane network, maximum reach of network comprising both bicycle lanes and off-road trails, and the time-invariant variable for commuting by bicycle in 1990. Estimated effects for changes for all time-varying variables were modeled by including a year* variable interaction term. ^b^Differences were obtained using the ‘margins’ post-estimation command following repeated-measures random effects linear regression models (-xtreg-) in Stata. ^c^ *P*-value for interaction = 0.06. ^d^Levels of predictor variables reflect the percentiles of the variable distribution for combined 2000 and 2010 data. For distance: 25^th^ = 1.08 km; 50^th^ = 2.83 km; 75^th^ = 5.91 km. For proportion of commuting trips that cross the trail: 25^th^ = 0.11; 50^th^ = 0.29; 75^th^ = 0.42

Our primary analysis focused on the additions of the Hiawatha Trail (4.7 miles) [21] and Midtown Greenway (5.5 miles) [22] which provide 10.2 miles of off-road paved paths transecting the city north-south and east-west, respectively, including a dedicated bicycle/pedestrian bridge over a busy freeway (Fig. 1). These trails connect residential neighborhoods to employment centers downtown and at the University of Minnesota. These vital trail system components were constructed between 2000 and 2007 [22] and provided substantial new opportunities for non-motorized transportation. The exposure was proximity to the new trails, measured as straight-line (Euclidean) distance between the centroid of each tract and the closest point of the interconnected trails. Among the 116 census tracts in this study, mean land area was 122.34 hectares (SD 82.90), ranging from 26.34 to 564.19 hectares.

To control for concomitant increases in other cycling infrastructure, data on Minneapolis on-road bicycle lanes were obtained from historical maps collected as part of ongoing work detailed elsewhere [23]. Briefly, we identified the existence of on-road bicycle lanes using maps produced during early (1999 and 2002) and late (2008 and 2011) years by a local cycling advocacy organization [24]. Total on-road bicycle lane length per tract was calculated as sum length of all bicycle lane segments attributed to each tract. We also calculated maximum reach of continuous, uninterrupted bicycle lane network traversable via cycling trips originating from the tract. The same two network measures were repeated for off-road paved trails and again with on-road bicycle lanes and off-road paved trails considered as a single, interconnected cycling-amenable network.

We used StreetMap Premium 2010 roads database (ESRI, Redlands, CA) to create measures of road network, by overlaying street data onto 2010 tract boundaries. We calculated intersection density as number of intersections in the tract divided by land area (km^2^), where intersection was defined as the junction point of 3 or more discrete street segments.

#### Commuters crossing the trails

Journey to work data from Census Transportation and Planning Products (CTPP) was used to estimate potential use of trails by commuters. CTPP Journey to Work data provides tabulations of tract-to-tract (origin to destination) travel flows of trips for work by employees age 16 and older. For each tract, we calculated the proportion of work-related trips that could potentially benefit from the Greenway/Hiawatha trail system infrastructure investment. Commuting trips were included in this analysis if the straight-line (Euclidean) connection between the origin and destination tract centroids intersected the Greenway/Hiawatha trail system. We considered these to be trips which might traverse the new trails for some portion of the commuting route, thus providing a measure of the extent that tract residents would benefit from the trails for commuting to work. For ease of interpretation, these trips are trips that can potentially cross the trail *compared to* trips which do not have this potential. Tract to tract data were only included up to the 99^th^ percentile of distance (11.1 miles). For sensitivity analyses, we created versions of the proportion of commuting trips variable restricted to commuting trips within: 1) 4-miles and 2) 8-miles; this helps address a potentially restricted commute distance for bicycles. In addition, we considered that commuting trips crossing both trail systems (at a diagonal) might benefit most from the new trails; therefore, we created a sensitivity variable limited to trips that crossed both trail systems. It is important to note that all variables derived from the CTPP reflect overall commuting trips, irrespective of commute mode.

#### Social and economic indicators

We used 2000 Census and 2008–2012 American Community Survey (ACS) tract-level data for social and economic personal and community indicators. We only selected covariates available from both census administrations including median household income, proportion of workers aged 18–34, proportion of the population with professional employment, and proportion of the population with a college degree. All Census data were harmonized to 2010 tract geography using Brown University’s LTDB methodology [20].

### Statistical analysis

The outcome of interest was change in proportion of workers who commuted by bicycle from 2000, before development of the two off-road trails, to 2010 (measured by ACS 2008–2012), after completion of the new off-road trail system. We conducted multivariable-adjusted repeated measures linear regression, with a random effect for tract, to model proportion of bicycle commuters relative to two exposures: 1) trail proximity measured as straight-line (Euclidean) distance from tract centroid to trails and 2) trail potential use measured as proportion of commuting trips to destinations across the trails. Midtown Greenway and Hiawatha Trail did not exist in 2000, so there is no distance variable or *p*-value for this date. We included interaction terms by year to test for trends (2010 vs 2000) in commuting by bicycle, and to control for trends in model covariates. In multivariable-adjusted regression, we controlled for tract-level time-varying population density, social and demographic covariates, total commuting trips, intersection density, total length of on-road bicycle lanes, maximum distance of bicycle lane networks accessible from the tract, maximum distance on combined bicycle lane and off-road trail network, and proportion of workers who commuted by bicycle in 1990. Specifically, we controlled for changes in tract-level socio-economic variables between 2000 and 2010, including changes in population density, median household income, the proportion of workforce professionally employed, the proportion of the workforce aged 18–34 years. We conducted sensitivity analyses with the alternate specifications of proportion of commuters crossing the trail system (as described above). These included regressions using the restricted commute distances (4-miles, 8-miles) and an analysis limited to trips that crossed both trail systems. All covariates were modeled continuously and tested for non-linearity. We considered the joint importance of the two exposures by testing the interaction between the variables. For ease of presentation, we calculate the 10-year change in bicycle commuting at the 25th, 50th, and 75th percentiles of each exposure. Percentiles for distance to trail were 1.08 (25^th^), 2.83 (50^th^), and 5.91 (75^th^) km, and percentiles for proportion of work-related trips that crossed the trail were 0.11 (25^th^), 0.29 (50^th^), and 0.42 (75^th^).

In addition, we examined the possibility of spatial autocorrelation in our data both for change in proportion of bicycle commuters between the two periods and for regression model residuals. In all analyses, neighbor tracts were defined using a row-standardized queen’s first order neighborhood matrix. For change, we used Getis-Ord General G, a Local Indicator of Spatial Association (LISA) in the High/Low Clustering function of ArcGIS 10.1’s Spatial Statistics package. Clusters were considered significant if they had a Z-Score of ≥1.96 or ≤ -1.96. Residuals from regression models were examined with Global Moran’s Index; no remaining spatial autocorrelation was detected, indicating spatial regression was unnecessary.

We used SAS (v.9.4), Stata/MP (v.14), and R for data management and statistical analysis, and ArcGIS to create geographic variables and produce displayed maps.

## Results

Percentage of workers who reported commuting by bicycle increased from 1.8% in 2000 to 4.0% in 2010 with an average 2010–2000 difference of 2.3% (Table 1). In contrast, the 2000–1990 difference in commuting by bicycle was 0.12%. There were not significant 2010 vs 2000 differences in total number of work-related trips or proportion of trips that potentially traverse the trails. Along with the addition of the off-road trails, there were significant increases in tract-level cycling infrastructure, including total length of bicycle lanes, and maximum network distances accessible on bicycle lanes alone and on both bicycle lanes and off-road trails. There were demographic changes, including increases in professional workforce relative to other job classes and proportion of the workforce aged 18 to 34.

**Table 1:** Tract^a^-level sociodemographic, neighborhood infrastructure, and commuting characteristics in Minneapolis, Minnesota, 2000 and 2010^b^

	2000	2010	
	Mean (SD)	Mean (SD)	*p*-value^c^
Commuting by bicycle^d^, %	1.76 (1.96)	4.04 (3.48)	<0.01
Change in cycling % from previous decade^d^	0.12 (1.7)	2.3 (3.0)	<0.01
Tract population, n	3,299 (1.18)	3,319 (1,299)	0.85
Workers aged 16+, n	1,792 (800)	1,766 (822)	0.73
Total commuting trips for work from tract^e^, n	1,530 (712)	1,526 (711)	0.97
Commuting trips from tract that cross the trail system^e^, %	0.27.4 (0.16.9)	27.6 (16.3)	0.95
Intersection density (3+ links, per sq km)^f^	59.9 (16.2)	70.4 (21.0)	<0.01
Total bicycle lane length, km^g^	1.69 (1.62)	2.88 (1.90)	<0.01
Maximum reach of bicycle lane network, km^g^	114 (60)	261 (50)	<0.01
Total bicycle trail length, km^g^	0.88 (1.65)	1.31 (2.10)	<0.01
Maximum reach of off-road trail network, km^g^	12.2 (15.7)	67.6 (63.1)	0.02
Maximum combined reach of bicycle lane and off-road trail network, km^g^	217.7 (87.5)	410.1 (38.4)	<0.01
Distance to trail system, km^h^	---	3.70 (2.95)	na
College graduate, % population > =25 years old	34.6 (19.7)	42.1 (20.5)	<0.01
Non-Hispanic white, % population	60.9 (26.6)	59.8 (25.9)	0.63
Professional employment, % workers	38.1 (14.7)	44.5 (16.5)	<0.01
Workers 18–34 years old, %	33.2 (12.6)	66.5 (28.7)	<0.01
Median household income, $10,000	5.52 (2.20)	5.20 (2.6)	0.14

^a^116 tracts harmonized to 2010 Census boundary delineations

^b^Tract-level 2010 Census data used unless noted

^c^Wilcoxon rank test comparing 2000 and 2010

^d^2010 variable derived from 2008 to 2012 pooled tract-level American Community Survey (ACS) data

^e^Data on commuting trips for work and destination obtained from the Census Transportation and Planning Products

^f^Intersection density was calculated as the number of intersections in tract divided by tract land area (km) based on the ESRI StreetMap Premium 2010 road database, with intersections defined as the junction of 3 or more street segments, excluding dead-ends and cul-de-sacs

^g^Bicycle lane and off-road trail data were from local sources as documented elsewhere [23]

^h^Distance to the trail system was the distance between the centroid of the tract and the closest point of the interconnected trail system. The Midtown Greenway and Hiawatha Trail did not exist in 2000, so there is no distance variable and no *p*-value

Tract-level commuting by bicycle was negatively associated with distance to the trails and positively associated with proportion of commuting trips crossing the trails; effect estimates weakened but remained statistically significant upon multivariable-adjustment (Table 2). In models including mutual adjustment for the two exposures, however, estimates further weakened and the trend in bicycle commuting was no longer significantly associated with tract distance to the trails. Results were not substantively different in sensitivity analyses with the distance restrictions (4- and 8-miles), although effect sizes were slightly larger (Additional file 1: Table S1). In addition, consistent with findings for the proportion of all commuting trips crossing the trail system, there were significant 10-year increases in bicycle commuting for trails with higher proportion of commuting trips crossing both trails, (Additional file 1: Table S1). Note that these analyses were limited to tracts within a comparable distance (4- or 8- miles, respectively) of the trail system, with 98 tracts within 4-miles of the trail system and 113 tracts within 8-miles of the trail system.

**Table 2:** Differences in bicycle commuting (%) in 2010 vs 2000^a^ by trail access and potential use^b^

	Percentile	Unadjusted	Multivariable-adjusted, model 1^c^	Multivariable-adjusted, model 2^d^
Distance from trail system (km)	25^th^ (1.08 km)	3.00 (2.28, 3.72)	2.54 (0.71, 4.38)	2.03 (0.13, 3.93)
	50^th^ (2.83 km)	2.50 (1.94, 3.06)	1.99 (0.20, 3.77)	1.88 (0.10, 3.66)
	75^th^ (5.91 km)	1.62 (0.95, 2.29)	1.01 (-0.90.00, 2.92)	1.62 (-0.41, 3.65)
	*p*-value, trend	<0.01	<0.01	0.63
Proportion of commuting trips crossing the trail system	25^th^ (0.11)	1.20 (0.6, 1.95)	0.73 (-1.14, 2.61)	0.95 (-1.01, 2.90)
	50^th^ (0.29)	2.37 (1.84, 2.90)	1.83 (0.07, 3.59)	1.89 (0.08, 3.69)
	75^th^ (0.42)	3.22 (2.52, 3.92)	2.63 (0.79, 4.46)	2.57 (0.53, 4.60)
	*p*-value, trend	<0.01	<0.01	0.06

^a^Differences were obtained using the ‘margins’ post-estimation command following repeated-measures random effects linear regression models (-xtreg-) in Stata

^b^Levels of predictor variables (a. distance from the trail system and b. proportion of work-related trips that cross the trail system.) reflect the 25^th^, 50^th^, and 75^th^ percentiles of the variable distribution for combined 2000 and 2010 data

^c^Regression models included either distance to trail system (analysis a) or proportion to work-related trips that cross the trail system (analysis b), and adjusted for time-varying and tract-level covariates: total work-related trips, intersection density, population density, median household income, professional workforce, workforce aged 18-34 years, total length of bicycle lanes, maximum reach of bicycle lane network, maximum reach of network comprising both bicycle lanes and off-road trails, and the time-invariant variable for commuting by bicycle in 1990. Estimated effects for changes for all time-varying variables were modeled by including a year*variable interaction term

^d^Regression models included both distance to trail system and proportion of work-related trips that cross the trail system, controlling for the same set of covariates as in 3

Analyses of the joint exposure effect revealed significant 10-year increases in bicycle commuting restricted to tracts that: (1) were close to the off-road trail system and (2) had a higher proportion of commuting trips that crossed the trails (Fig. 1, Additional file 1: Table S2). For example, between 2000 and 2010, among tracts at 25^th^ percentile of distance from the trails, for tracts at 25^th^ percentile of proportion of work-related trips crossed the trails, the percent of workers who commuted by bicycle did not increase (beta = -0.03 (95^th^ CI: -2.39, 2.33). By contrast, at the same distance from the trails, tracts at 75^th^ percentile of proportion of work-related trips crossing the trails experienced a 2.58% increase (95^th^ CI: 0.71%, 4.46%). Similarly, among tracts at a high proportion of work-related trips that crossed the trails, a significant increase in commuting by bicycle was observed among tracts closer to the trail system, but not among tracts far away.

**Fig. 2:**
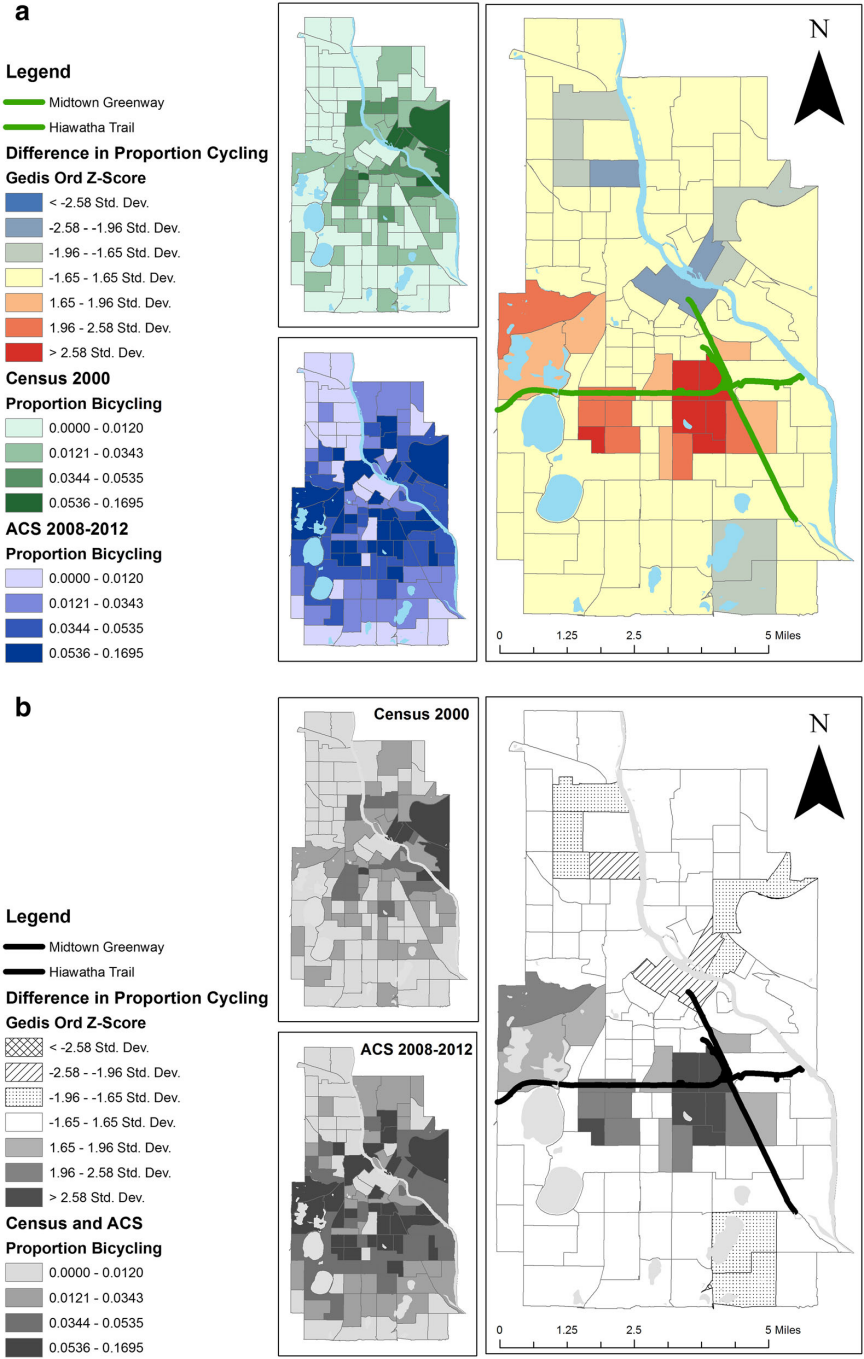
Location of new off-road trail infrastructure and spatial cluster analysis^a^ of proportion of commuting by bicycle in Census 2000 and American Community Survey (ACS) 2008–2012. ^a^ Clusters of difference in proportion commuting by bicycle identified using Getis - Ord General G, a Local Indicator of Spatial Association (LISA) and a row-standardized queen’s first order neighborhood matrix. Clusters should be considered significant if they have a Gedis Ord Z-Score of ≥1.96 or ≤ -1.96

Getis-Ord LISA statistics identified a cluster of high increase in proportion of population commuting by bicycle between Census 2000 and ACS 2008–2012 (Fig. 2). Neighborhoods in these high clusters tended to be located near off-road trails, with the most significant high increases in the proportion of population commuting by bicycle occurring at the intersection of the Midtown Greenway and Hiawatha Trail.

## Discussion

Our data indicate that development of an off-road trail system in Minneapolis between 2000 and 2007 contributed to significant increases in commuting by bicycle between 2000 and 2008–2012. Specifically, against a background of increased cycling nationally and within Minneapolis, commuting by bicycle increased even further among tracts that were close (approximately 3 km) to the trail. Higher bicycle commuting was also seen in tracts with a large proportion of work-related trips that crossed the trails. These results highlight the joint importance of proximity to off-road trails as well as underlying potential community travel patterns in relation to commuting behavior.

The observed increase in bicycle commuting with the implementation of new bicycle infrastructure is consistent with, and expands upon, previous literature on bicycle infrastructure and bicycle behavior [9, 11–17, 25–27]. Cross-sectional analyses showed associations between more bicycle infrastructure and higher prevalence of bicycle commuting behavior [9, 25]. Our longitudinal analysis gives further insight into the potential for bicycle infrastructure changes to influence changes in commuting behavior. Among the limited existing longitudinal research, results are mixed [11–17, 26, 27]. A study of 353 adults in Portland, Oregon found that new bicycle boulevards were not associated with increases in physical activity. One potential reason for the inconsistency between the Portland results and our findings might be the type of infrastructure investigated. Our analyses focused on two off-road paved trails, which have been shown to be preferred [28, 29]. In particular, off-road trails are thought to support increased commuting by underrepresented groups such as women [30]. The importance of off-road trails was supported by work in the United Kingdom that found increased activity after the addition of traffic-free routes for walking and cycling [16, 27]. Our work confirms previous longitudinal research in Minneapolis showing increases in bicycle commuting in areas near bicycle facilities between 1990 and 2000 [17]. We extend this research by examining the years 2000 and 2008–2012 and new investments in off-road infrastructure. Due to its size, density, and variety of neighborhoods, Minneapolis may be generalizable to many other U.S. contexts. However, our work contributes to a growing body of literature on bicycle infrastructure and impels further inquiries leveraging longitudinal data for natural experiments in other geographic settings and populations.

A number of strengths make our paper noteworthy. First, our paper addresses previous calls for natural experiments using longitudinal changes in infrastructure to assess potential impacts of urban planning interventions to increase active commuting [26, 31]. Second, we controlled for other changes that may impact decisions to commute by bicycle, including demographics and secular increases in cycling in Minneapolis over the same period. We also included on-street bicycle lanes, network reach (how far one can travel from the tract) and total length within the tract, three factors identified as important for bicycle commuting [32]. Controlling for these other factors that may increase bicycling behavior, allowed us to isolate the additional bicycling benefit from the Hiawatha Trail and Midtown Greenway. Third, we had a sufficient timespan to capture changes in behavior. Finally, by considering both distance to the trails and proportion of commuters traveling to destinations via the trail, we are able to observe the joint importance of proximity to the trail from residences and the overall trail placement relative to economic centers. Our findings stress the value of careful planning to consider current commute patterns when implementing off-road trails.

However, some limitations remain. First, we focused on commuting, a small percentage of overall bicycle trips [9]. In addition to the societal benefits of active commuting for health and city design [5], we did this because the design of these two off-road trails was conducive to commuting by linking residential areas with commercial and employment centers. Additionally, our focus on commuting allowed us to use CTPP data, which provides enumerations of pairwise tract-to-tract commuting flows and may be more representative than Minneapolis-area travel survey data, since the travel survey is based on a random sample of households from 19 counties and may comprise a relatively small proportion of individuals living within Minneapolis census tracts. A second major limitation is aggregate analysis, which may change based on size and scale of aggregation [33] and lacks individual level commuting behavior [34]. Third, we do not know whether people seek residential locations closer to the trail to use the trail or whether existing residents change their behaviors and, as an aggregate analysis, did not control for length of time in neighborhood. Fourth, our data only examine bicycle commuting and do not include changes that may occur to leisure-time bicycling which may also be heavily influenced by new infrastructure [18]. Finally, our analysis may be limited by unmeasured variables such as data on bicycle ownership or other infrastructure changes such as the introduction of a new light rail service in Minneapolis in 2004. Nonetheless, the current results suggest that off-road bicycle facilities may be important for increasing overall cycling levels.

## Conclusions

Given the importance of active commuting for health [4], building environments that facilitate active travel can be influential. Our work supports the Complete Streets policies [6] and the U.S. Surgeon General’s recent Call to Action for communities benefiting those who run, bicycle, skate, or use wheelchairs [7]. We find increased bicycle commuting in tracts closest to a new off-road trail system in Minneapolis. These increases are largest for tracts with travel patterns that cross the trails for commuting to work destinations. Our ability to leverage longitudinal data for a natural experiment lends strong evidence to the increases in bicycle commuting that could occur with focused investments from municipalities. Our work thus informs municipal decision-making relative to investment in cycling infrastructure, and highlights the potential health benefits of new urban planning infrastructure. Several unique features of Minneapolis trails, including separation from roads and optimized routes connecting residential and employment areas, may have increased their ability to encourage cycling. Ultimately, our findings support a maintained connection between public health practitioners, policy makers, urban planners, and communities as the U.S. strives to build healthy cities and encourage the shift to more active travel modes.

## Additional file

Additional file 1: Table S1. Sensitivity analysis of estimated differences in bicycle commuting (%) in 2010 vs 2000 by trail access and potential use. **Table S2.** Multivariable-adjusted^a^ regression estimates for the difference^b^ in the percentage of workers commuting by bicycle in 2000 and 2010 according to joint levels of the distance between the tract and the trail system^c^ and proportion of commuting trips that cross the trail system^c^. (DOCX 21 kb)

## Abbreviations

ACS: American Community Survey
CI: Confidence Interval
CTPP: Census Transportation Planning Products
LISA: Local indicators of spatial autocorrelation
LTDB: Longitudinal Tract Data Base
