# Supplementary material for: Municipal investment in off-road trails and changes in bicycle commuting in Minneapolis, Minnesota over 10 years: a longitudinal repeated cross-sectional study
Source: Int J Behav Nutr Phys Act. 2017 Feb 13;14:21. doi: 10.1186/s12966-017-0475-1 (PMC5307757; doi:10.1186/s12966-017-0475-1)
Supplement: Additional file 1: Table S1. — Sensitivity analysis of estimated differences in bicycle commuting (%) in 2010 vs 2000 by trail access and potential use. Table S2. Multivariable-adjusteda regression estimates for the differenceb in the percentage of workers commuting by bicycle in 2000 and 2010 according to joint levels of the distance between the tract and the trail systemc and proportion of commuting trips that cross the trail systemc. (DOCX 21 kb) [file 12966_2017_475_MOESM1_ESM.docx]

**Supplemental Materials:**

**Supplemental Table S1. Sensitivity analysis of estimated differences in bicycle commuting (%) in 2010 vs 2000 by trail access and potential use.**

| Distance of commuting trips limited to 99^th^ percentile (11.1 miles) | | | | | |
| --- | --- | --- | --- | --- | --- |
|  | Percentile | Unadjusted | Multivariable-adjusted, model 1^c^ | Multivariable-adjusted, model 2^d^ | Multivariable-adjusted, model 2^d^ |
| Distance from trail system (km) | 25^th^ (1.08 km) | 3.00 (2.28, 3.72) | 2.54 (0.71, 4.38) | 2.03 (0.13, 3.93) | 2.36 (0.48, 4.25) |
|  | 50^th^ (2.83 km) | 2.50 (1.94, 3.06) | 1.99 (0.20, 3.77) | 1.88 (0.10, 3.66) | 1.90 (0.06, 3.75) |
|  | 75^th^ (5.91 km) | 1.62 (0.95, 2.29) | 1.01(-0.90, 2.92) | 1.62 (-0.41, 3.65) | 1.09 (-1.05, 3.24) |
|  | p-value, trend | <0.01 | <0.01 | 0.63 | 0.10 |
| Proportion of commuting trips crossing the trail system, | 25^th^ (0.11) | 1.20 (0.46, 1.95) | 0.73 (-1.14, 2.61) | 0.95 (-1.01, 2.90) |  |
|  | 50^th^ (0.29) | 2.37 (1.85, 2.90) | 1.83 (0.07, 3.59) | 1.89 (0.08, 3.69) |  |
|  | 75^th^ (0.42) | 3.22 (2.52, 3.92) | 2.63 (0.79, 4.46) | 2.57 (0.53, 4.60) |  |
|  | p-value, trend | <0.01 | <0.01 | 0.07 |  |
| Proportion of commuting trips crossing both legs of the trail system | 25^th^ (0.011) | 1.77 (1.06, 2.47) | 1.20 (-0.64, 3.03) |  | 1.48 (-0.38, 3.35) |
|  | 50^th^ (0.061) | 2.15 (1.60, 2.70) | 1.58 (-0.22, 3.37) |  | 1.63 (-0.23, 3.48) |
|  | 75^th^ (0.11) | 2.53 (1.94, 3.12) | 1.96 (0.12, 3.79) |  | 1.77 (-0.21, 3.76) |
|  | p-value, trend | 0.03 | 0.05 |  | 0.58 |
| ^a^Differences were obtained using the ‘margins’ post-estimation command following repeated-measures random effects linear regression models (-xtreg-) in Stata.  ^b^Levels of predictor variables (a. distance from the trail system and b. proportion of work-related trips that cross the trail system.) reflect the 25^th^, 50^th^, and 75^th^ percentiles of the variable distribution for combined 2000 and 2010 data.  ^c^Regression models included either distance to trail system (analysis a) or proportion to work-related trips that cross the trail system (analysis b), and adjusted for time-varying and tract-level covariates: total work-related trips, intersection density, population density, median household income, professional workforce, workforce aged 13-34 years, total length of bicycle lanes, maximum reach of bicycle lane network, maximum reach of network comprising both bicycle lanes and off-road trails, and the time-invariant variable for commuting by bicycle in 1990. Estimated effects for changes for all time-varying variables were modeled by including a year**variable* interaction term.  ^d^Regression models included both distance to trail system and proportion of work-related trips that cross the trail system, controlling for the same set of covariates as in 3. | | | | |  |

| Distance of commuting trips limited to 8 miles | | | | | |
| --- | --- | --- | --- | --- | --- |
|  | Percentile | Unadjusted | Multivariable-adjusted, model 1^c^ | Multivariable-adjusted, model 2^d^ | Multivariable-adjusted, model 2^d^ |
| Distance from trail system (km)^e^ | 25^th^ (1.08 km) | 3.03 (2.29, 3.78) | 2.50 (0.63, 4.37) | 2.10 (0.14, 4.05) | 2.45 (0.53, 4.38) |
|  | 50^th^ (2.81 km) | 2.51 (1.94, 3.07) | 1.91 (0.09, 3.72) | 1.83 (0.1, 3.66) | 1.93 (0.04, 3.82) |
|  | 75^th^ (5.85 km) | 1.58 (0.84, 2.31) | 0.86 (-1.10, 2.83) | 1.41 (-0.75, 3.52) | 1.02 (-1.24, 3.28) |
|  | p-value, trend | <0.01 | <0.01 | 0.44 | 0.09 |
| Proportion of commuting trips crossing the trail system | 25^th^ (0.07) | 1.24 (0.46, 2.02) | 0.71 (-1.23, 2.64) | 1.02 (-1.01, 3.05) |  |
|  | 50^th^ (0.27) | 2.34 (1.81, 2.88) | 1.69 (-0.11, 3.49) | 1.74 (-0.10, 3.58) |  |
|  | 75^th^ (0.42) | 3.21 (2.50, 3.92) | 2.47 (0.60, 4.34) | 2.31 (0.24, 4.39) |  |
|  | p-value, trend | <0.01 | <0.01 | 0.17 |  |
| Proportion of commuting trips crossing both legs of the trail system | 25^th^ (0.006) | 1.79 (1.08, 2.50) | 1.29 (-0.58, 3.15) |  | 1.60 (-0.30, 3.49) |
|  | 50^th^ (0.043) | 2.08 (1.49, 2.66) | 1.54 (-0.30, 3.38) |  | 1.67 (-0.22, 3.55) |
|  | 75^th^ (0.11) | 2.60 (1.99, 3.20) | 2.00 (0.11, 3.88) |  | 1.79 (-0.26, 3.84) |
|  | p-value, trend | 0.02 | 0.07 |  | 0.72 |
| ^a^Differences were obtained using the ‘margins’ post-estimation command following repeated-measures random effects linear regression models (-xtreg-) in Stata.  ^b^Levels of predictor variables (a. distance from the trail system and b. proportion of work-related trips that cross the trail system.) reflect the 25^th^, 50^th^, and 75^th^ percentiles of the variable distribution for combined 2000 and 2010 data.  ^c^Regression models included either distance to trail system (analysis a) or proportion to work-related trips that cross the trail system (analysis b), and adjusted for time-varying and tract-level covariates: total work-related trips, intersection density, population density, median household income, professional workforce, workforce aged 13-34 years, total length of bicycle lanes, maximum reach of bicycle lane network, maximum reach of network comprising both bicycle lanes and off-road trails, and the time-invariant variable for commuting by bicycle in 1990. Estimated effects for changes for all time-varying variables were modeled by including a year**variable* interaction term.  ^d^Regression models included both distance to trail system and proportion of work-related trips that cross the trail system, controlling for the same set of covariates as in 3.  ^e^Analysis restricted to tracts within 8 miles (12.87 km) of the trail system. | | | | |  |

| Distance of commuting trips limited to 4 miles | | | | | |
| --- | --- | --- | --- | --- | --- |
|  | Percentile | Unadjusted | Multivariable-adjusted, model 1^c^ | Multivariable-adjusted, model 2^d^ | Multivariable-adjusted, model 2^d^ |
| Distance from trail system (km)^e^ | 25^th^ (1.01 km) | 3.06 (2.22, 3.89) | 2.67 (0.62, 4.73) | 2.19 (0.12, 4.27) | 2.50 (0.40, 4.59) |
|  | 50^th^ (2.24 km) | 2.69 (2.05, 3.34) | 2.23 (0.23, 4.21) | 2.16 (0.17, 4.14) | 2.19 (0.13, 4.24) |
|  | 75^th^ (4.91 km) | 1.90 (1.01, 2.80) | 1.26 (-0.89, 3.41) | 2.08 (-0.26, 4.41) | 1.52(-0.91, 3.94) |
|  | p-value, trend | 0.06 | 0.04 | 0.90 | 0.27 |
| Proportion of commuting trips crossing the trail system | 25^th^ (0.04) | 1.35 (0.47, 2.24) | 1.07 (-0.97, 3.10) | 1.09 (-1.04, 3.22) |  |
|  | 50^th^ (0.18) | 2.01 (1.34, 2.67) | 1.63 (-0.32, 3.59) | 1.65 (-0.35, 3.64) |  |
|  | 75^th^ (0.40) | 3.24 (2.54, 3.93) | 2.70 (0.70, 4.71) | 2.70 (0.56, 4.83) |  |
|  | p-value, trend | <0.01 | <0.01 | 0.04 |  |
| Proportion of commuting trips crossing both legs of the trail system | 25^th^ (0) |  |  |  |  |
|  | 50^th^ (0.01) | 2.16 (1.43, 2.88) | 1.76 (-0.23, 3.74) |  | 1.83 (-0.20, 3.85) |
|  | 75^th^ (0.081) | 2.57 (1.95, 3.19) | 2.16 (0.15, 4.17) |  | 2.06 (-0.04, 4.15) |
|  | p-value, trend | 0.05 | 0.08 |  | 0.44 |
| ^a^Differences were obtained using the ‘margins’ post-estimation command following repeated-measures random effects linear regression models (-xtreg-) in Stata.  ^b^Levels of predictor variables (a. distance from the trail system and b. proportion of work-related trips that cross the trail system.) reflect the 25^th^, 50^th^, and 75^th^ percentiles of the variable distribution for combined 2000 and 2010 data.  ^c^Regression models included either distance to trail system (analysis a) or proportion to work-related trips that cross the trail system (analysis b), and adjusted for time-varying and tract-level covariates: total work-related trips, intersection density, population density, median household income, professional workforce, workforce aged 13-34 years, total length of bicycle lanes, maximum reach of bicycle lane network, maximum reach of network comprising both bicycle lanes and off-road trails, and the time-invariant variable for commuting by bicycle in 1990. Estimated effects for changes for all time-varying variables were modeled by including a year**variable* interaction term.  ^d^Regression models included both distance to trail system and proportion of work-related trips that cross the trail system, controlling for the same set of covariates as in 3.  ^e^Analysis restricted to tracts within 4 miles (6.44 km) of the trail system. | | | | |  |

| **Table S2:** Multivariable-adjusted^a^ regression estimates for the difference^b^ in the percentage of workers commuting by bicycle in 2000 and 2010 according to joint levels of the distance between the tract and the trail system^c^ and proportion of commuting trips that cross the trail system^c^. | | |
| --- | --- | --- |
| Distance to trail system (km) | Proportion of commuting trips that cross the trail system | Difference in percentage commuting by bicycle (2010-2000) |
| 25^th^ Percentile (1.08) | 25^th^ Percentile (0.11) | -0.03 (-2.39, 2.33) |
|  | 50^th^ Percentile (0.29) | 1.49 (-0.39, 3.36) |
|  | 75^th^ Percentile (0.42) | 2.58 (0.71, 4.46) |
| 50^th^ Percentile (2.83) | 25^th^ Percentile (0.11) | 0.06 (-1.98, 2.10) |
|  | 50^th^ Percentile (0.29) | 0.95 (-0.86, 2.76) |
|  | 75^th^ Percentile (0.42) | 1.60 (-0.36, 3.55) |
| 75^th^ Percentile (5.91) | 25^th^ Percentile (0.11) | 0.22 (-1.67, 2.11) |
|  | 50^th^ Percentile (0.29) | 0.01 (-2.26, 2.28) |
|  | 75^th^ Percentile (0.42) | -0.14 (-3.13, 2.85) |
| ^a^Regression models included: time-varying and tract-level variables for distance to trail system, proportion to work-related trips that cross the trail system, total work-related trips, intersection density, population density, median household income, professional workforce, workforce aged 13-34 years, total length of bike lanes, maximum reach of bike lane network, maximum reach of network comprising both bike lanes and off-road trails, and the time-invariant variable for commuting by bicycle in 1990. Estimated effects for changes for all time-varying variables were modeled by including a year**variable* interaction term.  ^b^Differences were obtained using the ‘margins’ post-estimation command following repeated-measures random effects linear regression models (-xtreg-) in Stata.  ^c^Levels of predictor variables reflect the percentiles of the variable distribution for combined 2000 and 2010 data. For distance: 25^th^=1.08 km; 50^th^=2.83 km; 75^th^=5.91 km. For proportion of commuting trips that cross the trail: 25^th^=0.11; 50^th^=0.29; 75^th^=0.42. | | |
